# Dynamic Shear Deformation and Failure of Ti-6Al-4V and Ti-5Al-5Mo-5V-1Cr-1Fe Alloys

**DOI:** 10.3390/ma11010076

**Published:** 2018-01-05

**Authors:** Chun Ran, Pengwan Chen

**Affiliations:** State Key Laboratory of Explosion Science and Technology, Beijing Institute of Technology, Beijing 100081, China

**Keywords:** titanium alloys, split Hopkinson pressure bar, flat hat shaped specimen, digital image correlation technique, shear band

## Abstract

To study the dynamic shear deformation and failure properties of Ti-6Al-4V (Ti-64) alloy and Ti-5Al-5Mo-5V-1Cr-1Fe (Ti-55511) alloy, a series of forced shear tests on flat hat shaped (FHS) specimens for the two investigated materials was performed using a split Hopkinson pressure bar setup. The evolution of shear deformation was monitored by an ultra-high-speed camera (Kirana-05M). Localized shear band is induced in the two investigated materials under forced shear tests. Our results indicate that severe strain localization (adiabatic shear) is accompanied by a loss in the load carrying capacity, i.e., by a sudden drop in loading. Three distinct stages can be identified using a digital image correlation technique for accurate shear strain measurement. The microstructural analysis reveals that the dynamic failure mechanisms for Ti-64 and Ti-55511 alloys within the shear band are of a cohesive and adhesive nature, respectively.

## 1. Introduction

The term “adiabatic shear band” (also called “adiabatic shear” or “adiabatic slip”, hereinafter referred to as “ASB”) has been widely accepted by researchers since it was first mentioned in the original report of Zener and Hollomon in 1944 [1]. ASBs are found in different dynamic loading processes, such as impact deformation, dynamic punching, ballistic impact and so forth [2]. Shear localization is an important failure mechanism of solid materials in high strain rate deformation, for example, titanium alloys.

Titanium alloys have been extensively utilized for critical structural components in aerospace and automotive industries due to their highly attractive properties, such as high strength-to-weight ratio, good hardenability and excellent corrosion resistance [3]. A considerable number of investigations on titanium alloys under dynamic loading condition have been conducted over the last two decades [4,5,6], especially for Ti-6Al-4V (Ti-64) alloy. The works of Grebe et al. [7] and Bai et al. [8] pointed out that elliptical and spherical cavities were observed with the ASBs in Ti-64 alloy, and void coalescence resulted in elongated cavities. Peirs et al. [9] investigated the high strain rate shear behavior of Ti-64 alloy by using a hat shaped specimen and pointed out that the width of the shear region mainly affected the homogeneity of stress and deformation in the shear zone. The investigation of Xue et al. [10] indicated that the main stage of void evolution within shear bands in Ti-64 alloy were nucleation, growth and coalescence.

Increasing attention has been paid to Ti-5Al-5Mo-5V-1Cr-1Fe (Ti-55511) alloy over the past decade due to 15–20% weight reduction as compared to Ti-64 alloy [11]. Most of them focused on material aspects [12,13,14], while some of them focused on the mechanical properties under low strain rate loading conditions (<100 s^−1^) [15,16], and only a few number of publications reported the dynamic mechanical properties of Ti-55511 alloy [17].

Although the main mechanism for adiabatic shearing is the competition between hardening effect (strain and strain rate) and thermal softening effect, the whole process is very complex and involves high strain rates, high local temperature, large plastic deformation and so on. Unfortunately, the evolution of shear strain in shear regions is seldom reported in the literature. Therefore, it is important to understand the whole ASB process since it is known as a precursor to failure or fracture.

The goals of this work are to obtain insights into (a) dynamic mechanical behavior of Ti-64 and Ti-55511 alloys and (b) the evolution of the strain field in the deformation gage section in real-time.

The paper is organized as follows: First, the experimental techniques are introduced briefly, including materials, specimen geometry, split Hopkinson pressure bar (SHPB) technique, digital image correlation (DIC) technique and high-speed temperature recording system (IR system). Next, experimental results and discussion are presented followed by concluding remarks.

## 2. Materials and Experimental Techniques

### 2.1. Materials

Two titanium alloys were selected for this study: commercial Ti-64 and Ti-55511 [18], both supplied as extruded rods in the as-received condition. The *β* transus temperatures for Ti-64 and Ti-55511 alloys are approximately 980 and 860 °C, respectively. It should be pointed out that the flat hat shaped (FHS) specimens were all machined from the as-received bars.

### 2.2. Specimen Geometry

**Figure 1:**
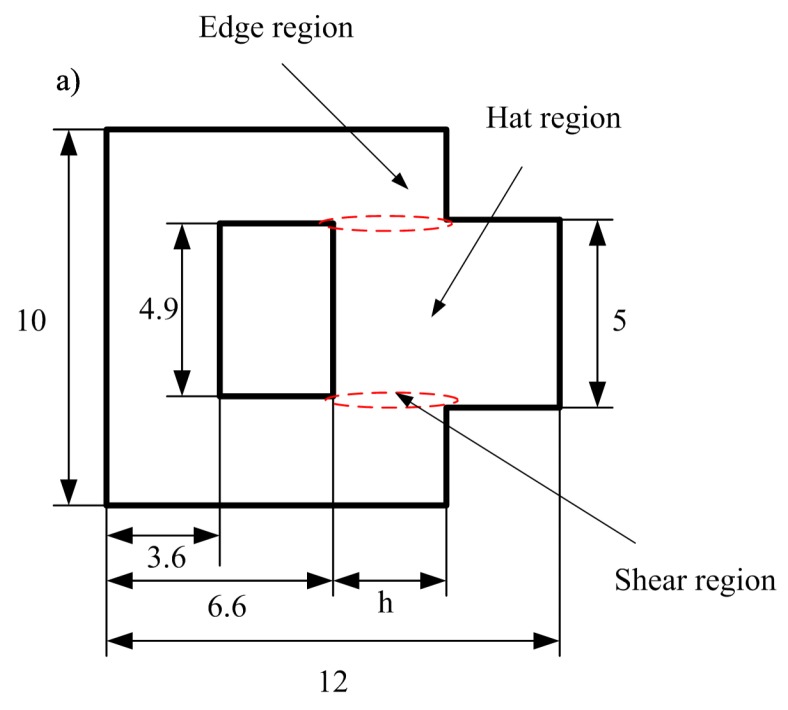

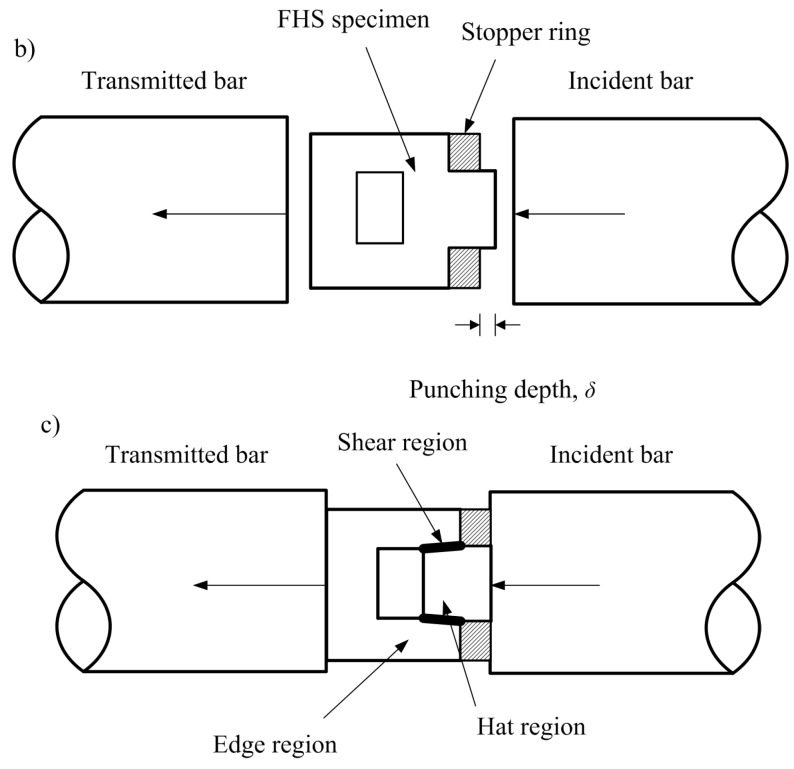
(**a**) In-plane dimensions of FHS specimen used in this study (dimensions in mm); (**b**) longitudinal section of FHS specimen, stopper ring, and dynamic testing configuration in SHPB setup; (**c**) specimen after testing with concentrated shear region. It should be pointed out that the values of *h* are equal to 3 and 2 mm for Ti-64 alloy and Ti-55511 alloy, respectively. The thickness of the FHS specimen for the two investigated materials is equal to 6 mm.

To reduce edge effect of hat shaped specimen and measure the temperature in the deforming gage section in real-time during the plastic deformation, the FHS specimen was designed by Clos et al. [19,20,21] and was successfully used in the study of large strain, high strain rate deformation of metals in conditions of forced localized shear [22]. The in-plane dimensions of the FHS specimen used in this study is illustrated in Figure 1a, specimens before and after forced shear tests are illustrated in Figure 1b,c, respectively. Compared to axi-symmetric where localization occurs over 360 degrees, Figure 1 shows planar concentrated deformation in the FHS specimen. As shown in Figure 1, the specimen can be divided into three parts: hat region, edge region, and shear region in which forced localization develops, and the designed thickness of shear region is equal to 0.1 mm (5 − 4.9).

### 2.3. Experimental Setup

**Figure 2:**
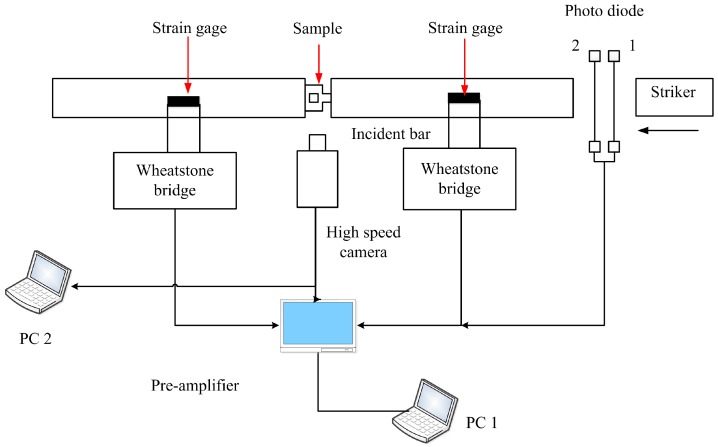
Schematic representations of the shear strain measurement device for a forced shear experiment.

Figure 2 is a schematic illustration of the experimental setup used in this study. It comprises two main parts: Standard SHPB technique and DIC technique, for dynamic loading and superficial strain field determination, respectively. The forced shear tests were carried out at 20 °C by means of SHPB technique.

#### 2.3.1. SHPB Technique

The SHPB technique is used to load the specimens dynamically. The FHS specimen is sandwiched between incident and transmission bar (Figure 2). An impact pulse is provided with a striker driven by a light gas gun. It should be noted that the setup used in this work is made of hardened 18% nickel maraging steel bars with diameters of 14 mm and the lengths of the striker, incident and transmission bar are 0.3 m, 1.2 m and 1.2 m, respectively. It should be pointed out that bar-specimen interfaces are sufficiently lubricated in order to reduce friction and specimen barreling, and three FHS specimens were used to test forced shear properties under each loading condition.

The velocity of the striker bar, *v*, can be estimated as the distance between the photo diodes (*d*) divided by the time (*t*) recorded by data acquisition instrument. When one-dimensional stress-waves in the bars are achieved and the specimen is in a state of uniform stress, the total axial loading history in the specimen can be determined by [23].

(1) $$F=E_{0}A_{0}\varepsilon_{t}\left(t\right)$$

where *E*_0_ is the Young’s Modulus of the Hopkinson bars, *A*_0_ is the cross-section area of the bars, and *ε_t_*(*t*) is the strain produced by transmitted wave.

The stress in the shear region of the FHS specimen can be approximated as follows. Assuming that *F* is uniformly distributed, then, *F* can be considered as shear part (*F_y_*) and compression part (*F_x_*). Since the overlapping distance is about 0.1 mm, which is very small compared to the thickness of the deformed section, so the angle of the sloped shear section (*θ*) is very small. Then, the components of *F* and the area of the shear section (*A*) can be expressed as:

(2) $$F_{x}=F\sin\theta \approx F\theta =F\frac{\left(a-b\right)}{2h}$$

(3) $$F_{y}=F\cos\theta \approx F$$

(4) $$A=\sqrt{{\left(a-b\right)}^{2}+h^{2}}\times t_{h}\approx h\times t_{h}$$

where *t_h_* is the thickness of the FHS specimen.

Then, the shear stress, *τ*, of the specimen can be approximated by:

(5) $$\tau \approx \frac{\pi E_{0}r_{0}^{2}\varepsilon_{t}\left(t\right)}{2ht_{h}}$$

where *r*_0_ is the radius of the Hopkinson bars.

The punching depth (displacement), *δ*, can be estimated approximately as

(6) $$\delta =-2C_{0}\int_{0}^{t}\varepsilon_{r}\left(t\right)dt$$

where *C*_0_ is the elastic bar wave speed in the bar material, and *ε_r_*(*t*) is the reflected strain histories in the bars at the specimen ends. Here, *C*_0_ = 4900 m/s.

Note that the shear strain evolution is very complex due to the evolution of the width of the shear region, and this will be addressed next.

#### 2.3.2. DIC Technique

DIC technique is a noncontact optical method and can measure the full-field strain distribution over a sample surface. Through mathematical contrasting the sub-images between deformed and un-deformed images, the deformation of the samples can be calculated [22,24]. It has been repeatedly used in Sutton et al. [25], Yan et al. [26], Chen et al. [27] and White et al. [22] to monitor the evolution of the shear strain in the deformation region in real-time during the deformation period. The strain field of the FHS specimen during the deformation process is calculated by 2D-DIC (VIC-2D. Correlated Solutions Incorporated, 120 Kaminer Way Parkway Suite, Columbia, SC 29210, USA, www.correlatedsolutions.com) technique.

The typical outline for acquiring full-field deformations of specimens is as follows:

- To obtain a random black-and-white speckle pattern with a spatial variation in intensity, the specimen was lightly coated, which was appropriate for displacement measurement using computer vision. To improve the bonding between the coating and the specimen, commercial spray paints (matt white and matt black) were adopted for the speckle pattern on all specimens.
- Grease was used to stick FHS specimen between the incident and transmission bar, and the ultra-high-speed camera, Kirana-05M (Kirana-05M. Specialised Imaging Ltd., Unit 32 Silk Mill Industrial Estate, Brook Street, Tring HP23 5EF, UK, www.specialised-imaging.com.cn), was located normal to the surface of the specimen.
- Two halogen lamps with a power of 1 kilowatt were used as the lighting source. The framing rate of the camera is 1,000,000 frames per second and the image resolution is 924 × 768 pixels.

The stress wave reached the forehead of the FHS specimen 550 μs later since the forehead of the striker passed through photo diode 1. Because only 180 frames can be saved in the ultra-high-speed camera, to avoid losing important deformation information during the punching process, the ultra-high-speed camera was triggered 530 μs later since the forehead of the striker passed through photo diode 1.

Note here that while the shear stress could be approximated using Equation (5), the shear strain was directly measured using DIC technique, as explained in the sequel.

## 3. Results and Discussion

A series of stopper rings were used to limit the deformation in the punching process. Different nominal shear strains were obtained by varying the thickness of the stopper ring. The stopper rings used in the present work were made of high strength steel to make sure only elastic deformation occurred.

### 3.1. Mechanical Response

**Figure 3:**
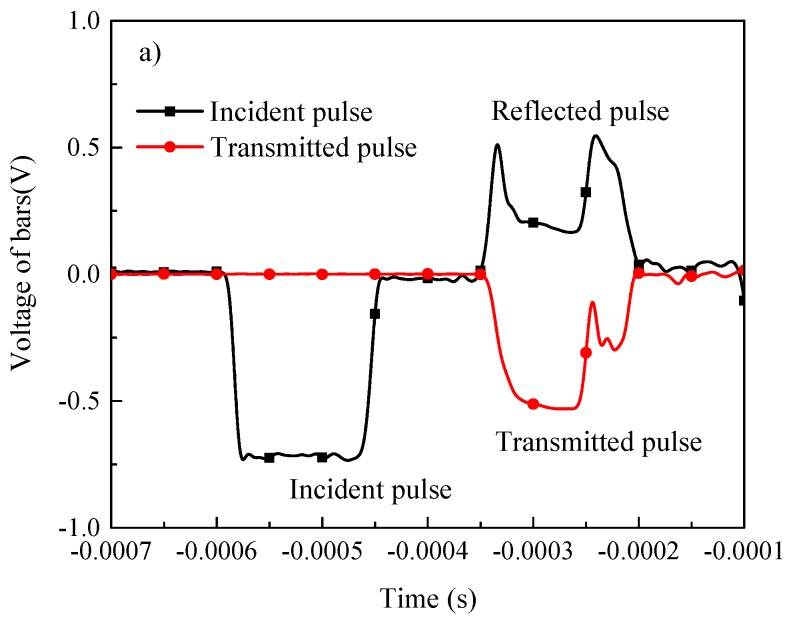

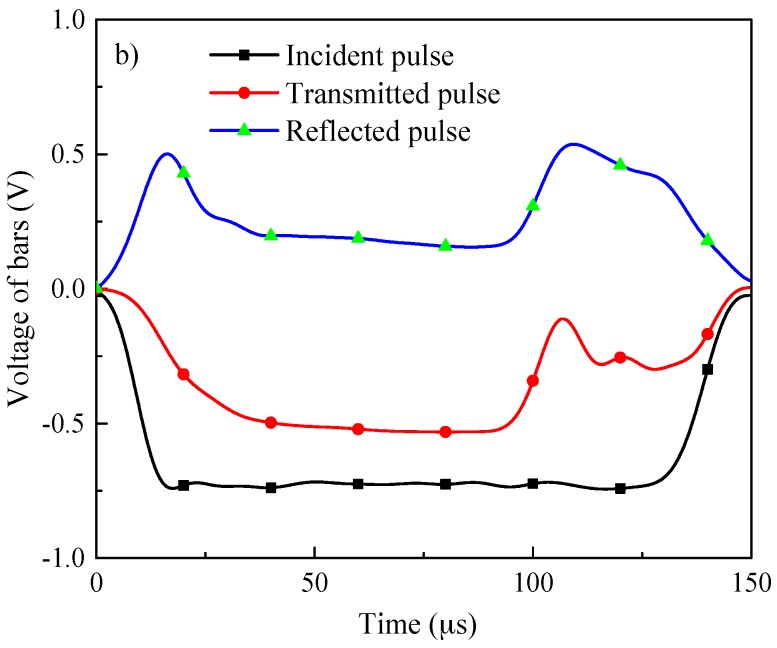
(**a**) Typical strain gage signals recorded in SHPB test at room temperature; (**b**) recorded signals after initiation to zero for elastic waves analysis.

Typical strain gage signals recorded in SHPB test and the signals after initiation to zero for elastic waves analysis are shown in Figure 3a,b, respectively. The punching depths, maximum loadings, maximum shear stresses, velocities of the striker bar, and dynamic failure energies are listed in Table 1.

**Figure 4:**
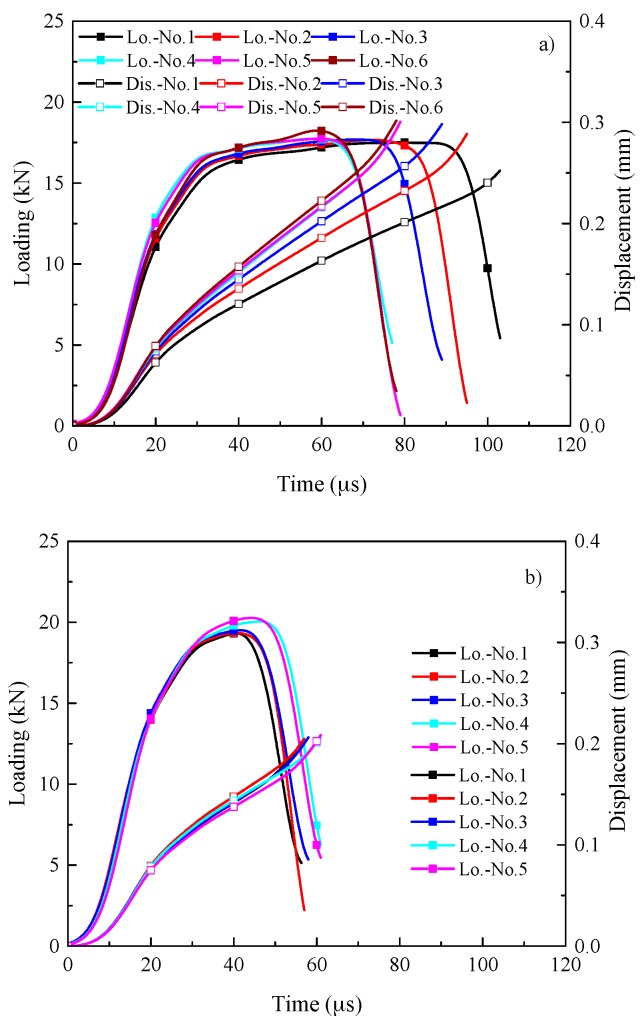
Loading and displacement vs. time for (**a**) Ti-64 alloy and (**b**) Ti-55511 alloy.

Figure 4 depicts the loading and displacement versus time for Ti-64 and Ti-55511 alloys. The results here all displayed using a similar scale to allow for comparison. As illustrated in Figure 4a, when the velocity of the striker bar maintains constant (9.1 m/s), the duration of shear deformation decreases with the increasing punching depth; for example, samples No. 2 and No. 3; when the punching depth maintains constant (0.3 mm), the loading/shear stress increases with increasing velocity of the striker bar; for instance, samples No. 2 and No. 4. In comparison with Ti-64 alloy, similar results for Ti-55511 alloy can be obtained. However, there still are some differences. The loading/shear stress of Ti-55511 alloy is higher than that of Ti-64 alloy, while the duration of plastic deformation is lower than that of Ti-64 alloy.

### 3.2. Shear Strain Measurement and Analysis

**Figure 5:**
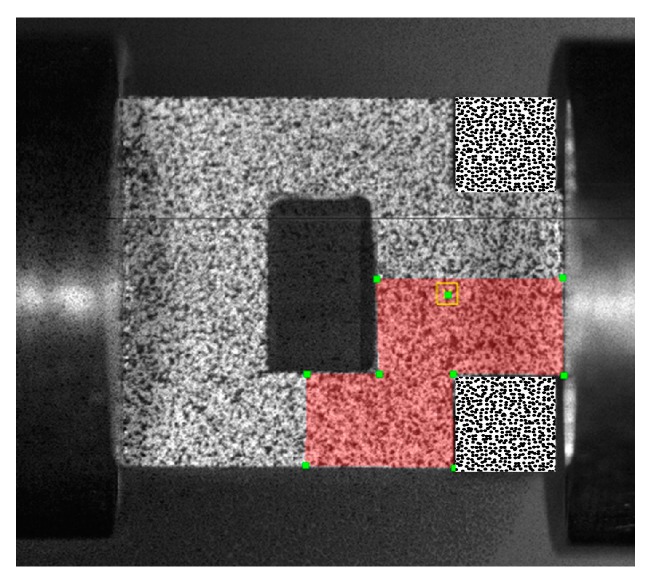
Diagram of speckle computing area.

**Figure 6:**
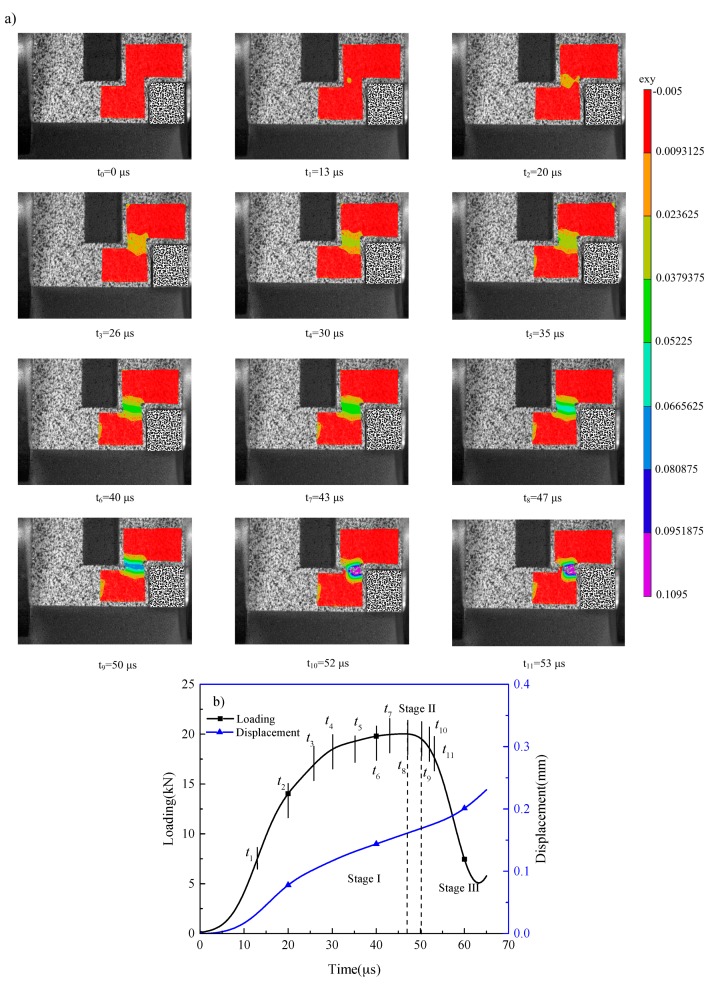
Tensorial shear strain field evolution of Ti-55511 alloy loaded at 9.2 m/s: (**a**) strain field at different time; (**b**) loading and displacement vs. time.

The diagram of speckle computing area is shown in Figure 5. A polygon area was selected as the AOI (area of interest) to analyze the deformation fields. To calculate accurately, the subset size of the correlation calculation was 17 × 17 pixels with a step size of 2 pixels. Typical tensorial shear strain (*ε_xy_*) field evolution within the shear region of Ti-55511 alloy is shown in Figure 6a, and the corresponding loading and displacement versus time curves are illustrated in Figure 6b. The value of engineering shear strain (*γ_eng._*) is twice the magnitude of *ε_xy_* measured by DIC technique [24], With the development of punching depth, the deformation within the shear region becomes increasingly larger, which may lead to some speckle failure to calculate the strain field. Moreover, the higher values of shear strain, the worse the speckle failure within the band. Thus, as shown in Figure 6a, the shear strain of Ti-55511 alloy deformed at 9.2 m/s, and measured by DIC technique, is not accurate beyond *t* ≥ *t*_9_. However, the trend of strain development can be obtained through the variations of the images. It should be pointed out that the shear strain is measured up to the maximum load for Ti-64 alloy.

Marchand and Duffy [28] and Rittel and Wang [29] defined three distinct stages of ASB formation. As shown in Figure 6, the similar three stages can be identified in the present work.

**Figure 7:**
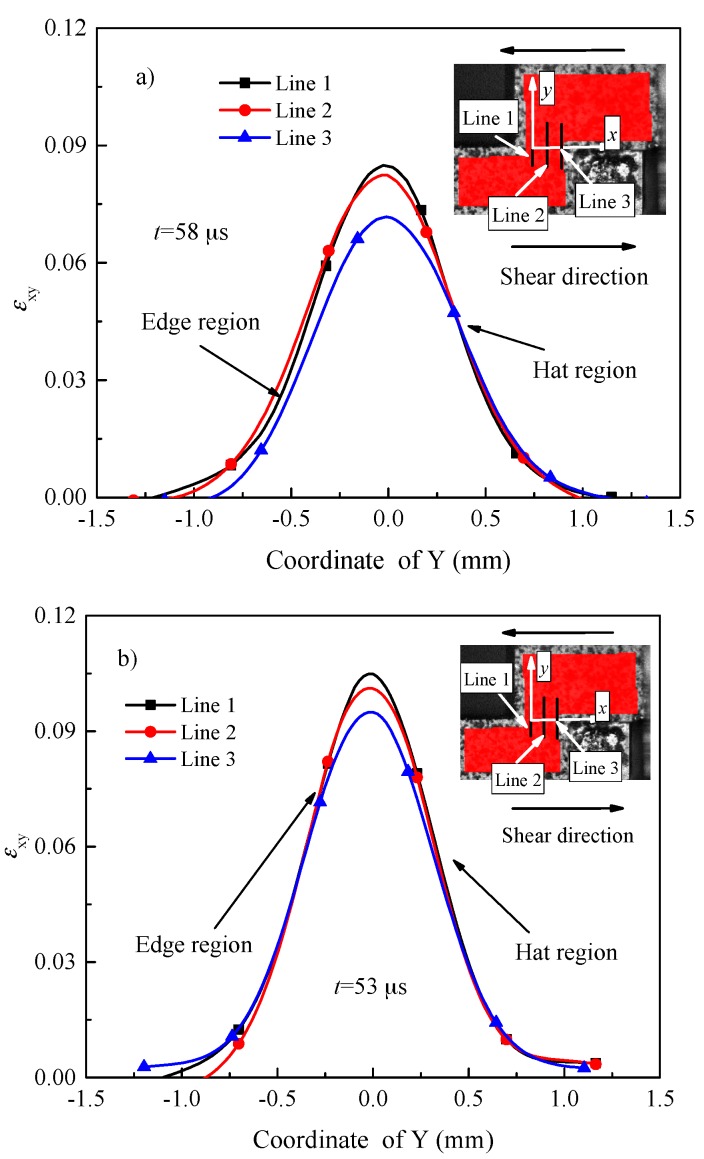
Distribution of shear strain for different positions along the shear region for (**a**) Ti-64 alloy at 58 μs, and the velocity of the striker bar is equal to 9.2 m/s; (**b**) Ti-55511 alloy at 53 μs, and the velocity of the striker bar is equal to 9 m/s. The center of shear region is defined as *x* axis, and *y* axis is perpendicular to the shear region.

**Figure 8:**
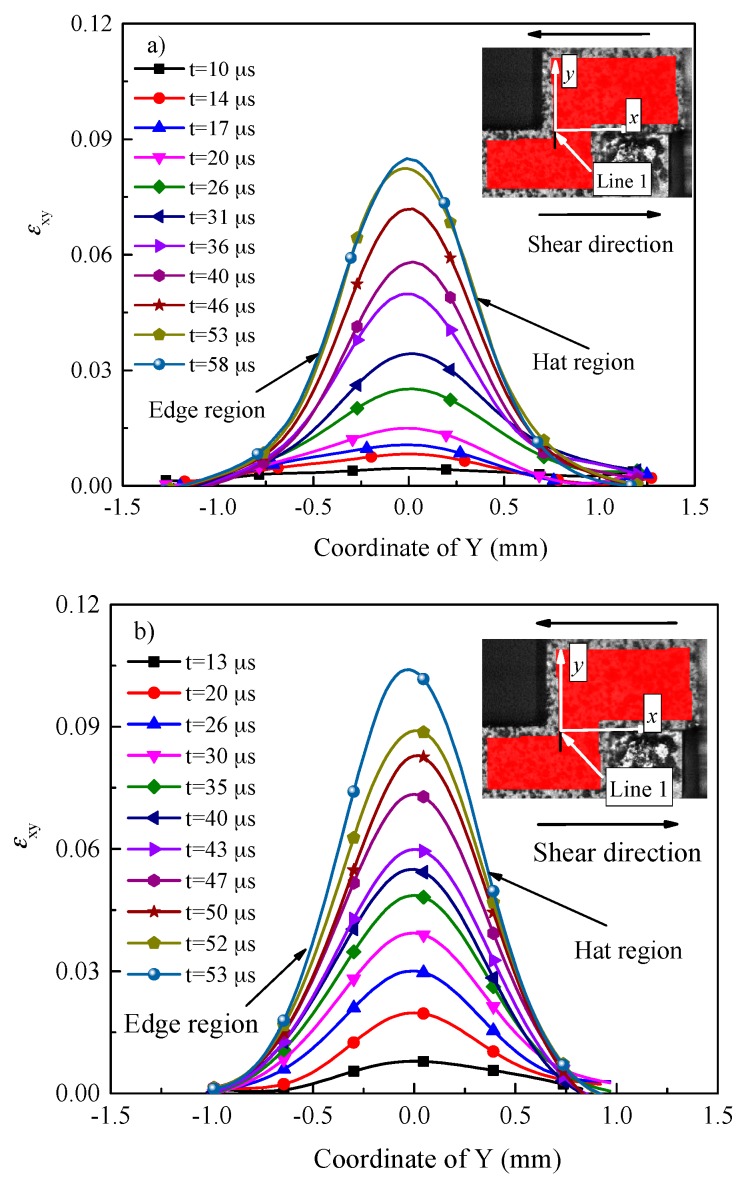
Distribution of shear strain at line 1 at different loading time for (**a**) Ti-64 alloy deformed at 9.2 m/s; and (**b**) Ti-55511 alloy deformed at 9 m/s. The center of shear region is defined as *x* axis, and *y* axis is perpendicular to the shear region.

**Figure 9:**
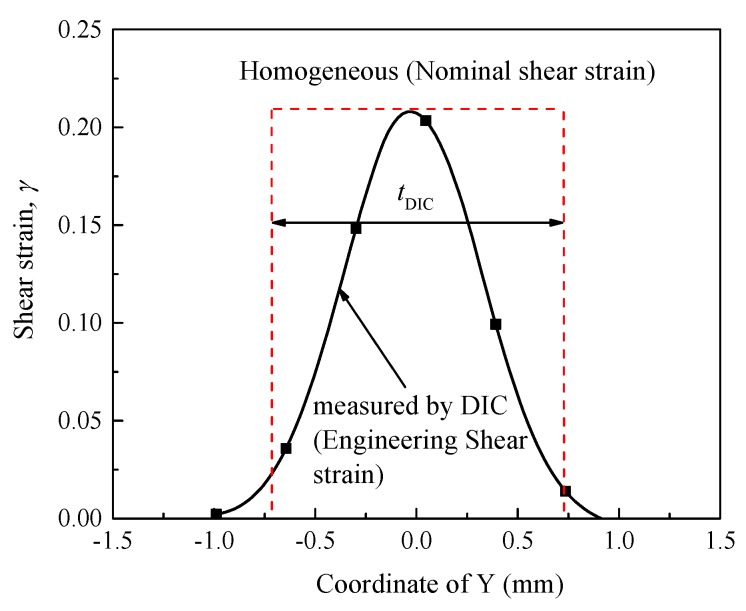
Schematic representations of nominal shear strain calculation.

Stage 1: This stage extends from *t* = *t*_0_ to *t* = *t*_8_ (*F_max._*). Strain concentration occurs at the left end of the shear region (close to the hole) when *t* = *t*_1_. A homogeneous shear strain concentrated band occurs when *t* = *t*_2_, and the thickness of the shear strain concentrated band increases to the maximum value (ca. 1.4 mm, see Figure 7, Figure 8 and Figure 9) when *t* = *t*_3_. The value of *F* reaches the maximum (20.03 kN) when *t* = *t*_8_, and a higher and narrower shear strain concentrated band (cyan color) occurs. Hence, homogeneous shear deformation occurs in this stage.
Stage 2: Starts from the time that corresponds to the maximum loading (shear stress) to the time before the loading (shear stress) drastically drops (*t*_8_ < *t* < *t*_9_). During this stage, severe shear strain localization (blue color) nucleates and propagates within the center of the shear region. Referring to Marchand and Duffy [28] and Rittel and Wang [29], the second stage can be identified as that of “heterogeneous deformation”, preceding (full) localization. Though the deformation is not strictly homogeneous in this stage, it is not yet fully localized.
Stage 3: This stage corresponds to a sharp loading (shear stress) drop (*t* = *t*_9_). *t* = 50 μs and *t* = 52 μs reveal that strain localization increases with increasing nominal shear strain. From *t* = *t*_9_, the nominal shear strain is no longer representing the true status of the deformation gage as a result of much severer strain localization, indicating that ASB forms fully and propagates within the shear region in this stage.

Marchand and Duffy [28], Clos et al. [19], and Rittel and Wang [29,30] pointed out that the drastic drop in load carrying capacity was caused by initiation and propagation of adiabatic shear band. While additional work of Duffy’s group [31,32,33] showed that the drastic drop only corresponded to crack propagation along the shear band and the ASB formed far before the loading/stress drop. Based on the evolution of shear strain, the reason of decreasing load-carrying capacity for Ti-64 and Ti-55511 alloys comes from severe strain localization.

The shear region is divided into three parts evenly by three lines to better understand the distribution of tensorial shear strain along the shear direction. Line 1 is coincidence with *y* axis, and line 3 is at the end of the right side of the shear region. As shown in Figure 7, for the two investigated materials, the tensorial shear strain decreases gradually from line 1 to line 3, implying the largest deformation of the shear region is on the left side, namely, shear localization and fracture propagate from line 1 to line 3 (shown in Figure 6a). Concentrated strain initiates on the left side of shear region may come from the machines groove leaved by sample machining process.

Figure 8 depicts the distribution of shear strain on line 1 at different loading times for the two investigated materials. As shown in Figure 8, for the two investigated materials, the values of shear strain at the same location of the shear region increases with time due to the punching depth (displacement) increasing. It is interesting to note that the values of shear strain in the edge region are almost equal to that in the hat region at the same time. This phenomenon can be interpreted as similar plastic deformation occurs in the edge and hat regions, which is in contradiction with the findings reported by Ran et al. [17], implying that the edge effect of hat shaped specimens can be reduced using FHS specimens [19].

As shown in Figure 7 and Figure 8, the thickness of plastic shear region (*t*_DIC_) is approximately 1.4 mm for the two investigated materials, which is much higher than that of the designed one (0.1 mm, see Section 2.2). Hence, it seems unreasonable to use the designed thickness of shear region to calculate the shear strain (punching depth divided by the thickness of the shear region, as proposed by Meyers’ group [4,34]). In fact, this is widely used to calculate the shear strain of hat shaped specimens, which most likely leads to much higher values of the shear strain.

Figure 9 depicts the schematic plots of nominal shear strain calculation used in the present work. As shown in Figure 9, we substitute *t*_DIC_ = 1.4 mm for the designed one (0.1 mm). Then, nominal shear strain, *γ_nom._*, can be estimated approximately as:

(7) γnom.=δtDIC

**Figure 10:**
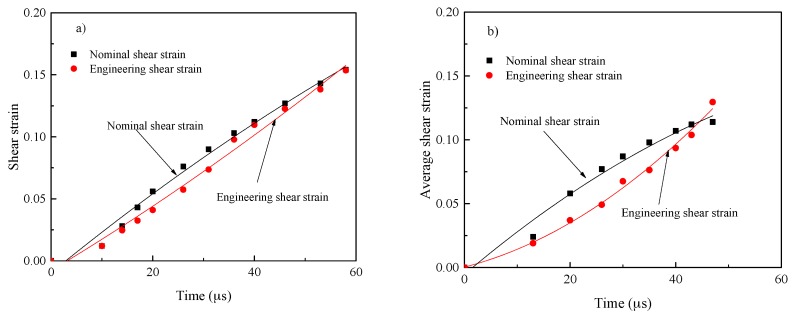
Plot of shear strain as a function of time for (**a**) Ti-64 alloy; and (**b**) Ti-55511 alloy.

Shear strain versus time for Ti-64 and Ti-55511 alloys are illustrated in Figure 10a,b, respectively. From Figure 10, it can be inferred that the nominal shear strain looks grossly averaged, and for all purposes, and the DIC data is more accurate. Figure 10 exemplifies the nature of the simplification achieved in calculations according to *t*_DIC_.

The engineering strain rate (${\dot{\gamma }}_{eng.}$) can be approximately estimated as the measured engineering shear strain divided by time. Based on Meyers’s group’s work [4,34], the strain rate ($\dot{\gamma }$) can be approximately estimated as half of the velocity of the striker bar divided by the thickness of the plastic deformation region. As mentioned above, the designed thickness of the plastic deformation region is much lower than that measured by DIC technique. Hence, the measured thickness was substituted for the designed one in the present study. The loading (*F*), punching depth (*δ*), nominal shear strain converted by SHPB technique (*γ_nom._*) and engineering shear strain measured by DIC technique (*γ_eng._*) at each moment for Ti-64 and Ti-55511 alloys are listed in Table 2 and Table 3, respectively.

**Figure 11:**
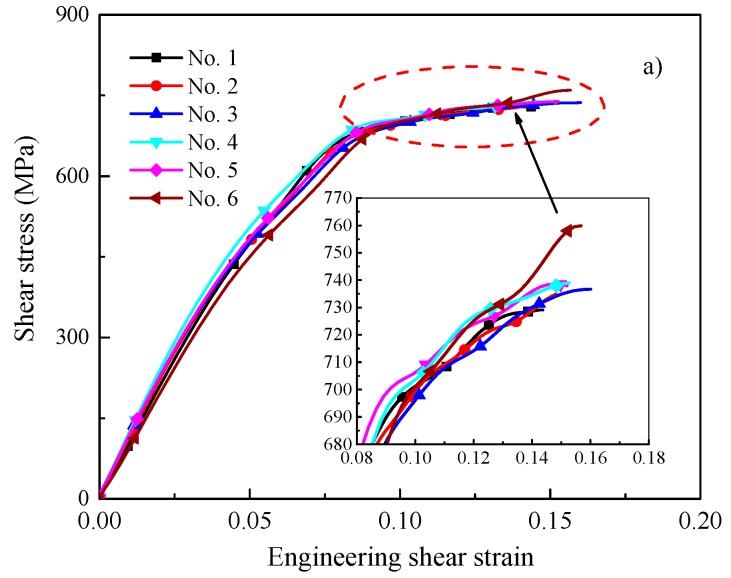

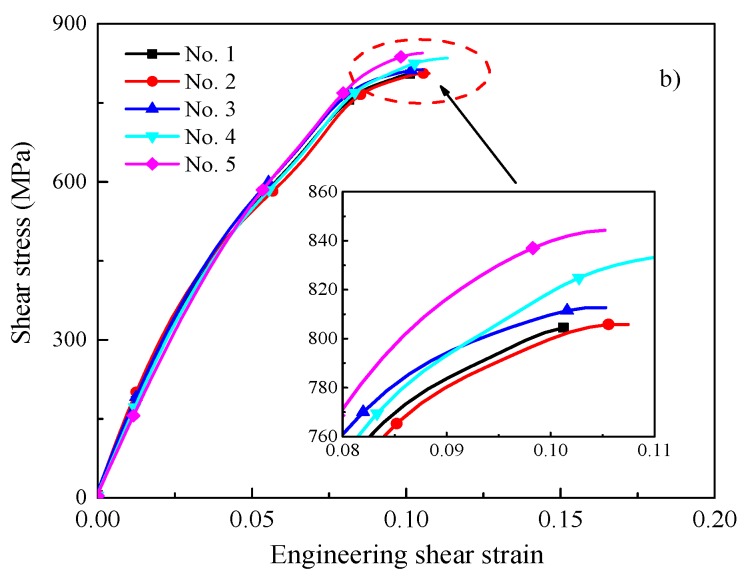
Plot of shear stress as a function of nominal shear strain for (**a**) Ti-64 alloy; and (**b**) Ti-55511 alloy.

The shear stress-engineering shear strain (measured by DIC) for Ti-64 alloy and Ti-55511 alloy are illustrated in Figure 11a,b, respectively. The small graphs inserted in the figures are the higher magnification of the dashed circle. As illustrated in Figure 11, the shear stress of Ti-55511 alloy is ca. 100 MPa higher than that of Ti-64 alloy, while the failure strain is lower than that of Ti-64 alloy. Simultaneously, the duration of plastic deformation of Ti-55511 is shorter than that of Ti-64 alloy (see Figure 4), implying that the plasticity of Ti-55511 alloy is inferior to Ti-64 alloy.

Based on Rittel et al. [35], the dynamic failure (mechanical) energy density, W, can be calculated as

(8) $$W=\int_{0}^{\gamma_{FS}}\tau d\gamma$$

where *γ_FS_* is the engineering failure strain (identified as the vicinity of the decreasing loading/stress stage). The forced shear experimental results have been listed in Table 4.

As shown in Figure 11, we can obtain that the maximum values of the failure strains for the two investigated materials increase with increasing the velocity of the striker bar, and it is not constant. Hence, the instability criterion used by Recht [36] and Culver [37] may be not useful for the two investigated materials.

**Figure 12:**
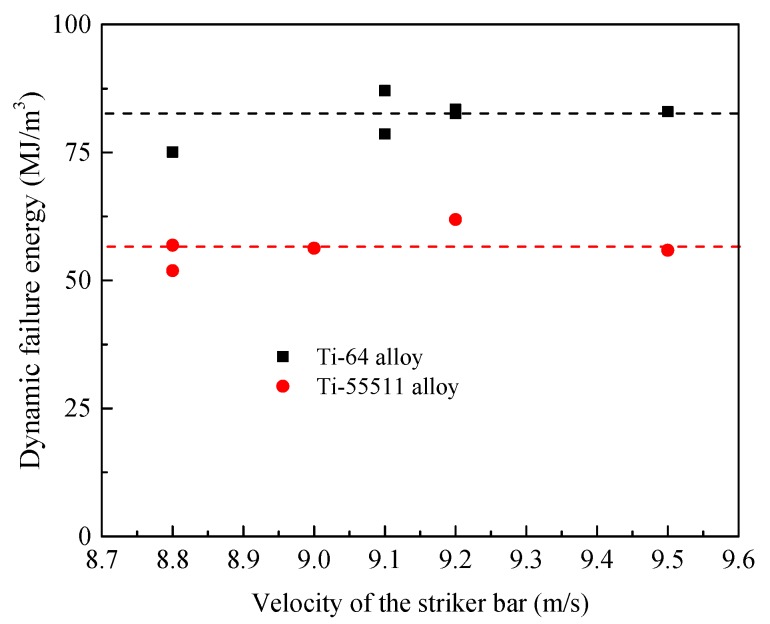
Plot of the dynamic failure (mechanical) energy of Ti-64 and Ti-55511 alloy as a function of the velocity of the striker bar.

Figure 12 shows the dynamic failure energy versus the velocity of the striker bar for the two investigated materials. As shown in Figure 12, the dynamic failure energy remains almost constant for Ti-64 and Ti-55511 alloys. By contrast, dynamic failure energy seems to represent a true material’s property due to its constant value. This is similar to the property of AM50, one kind of magnesium-aluminum alloy, and Ti-64 alloy (annealed condition), which was reported by Rittel et al. [35,38]. As shown in Figure 12, the dynamic failure energy of Ti-64 alloy is much higher than that of Ti-55511 alloy. Therefore, Ti-64 alloy is tougher than that of Ti-55511 alloy.

As shown in Figure 11, the maximum shear stress for Ti-64 and Ti-55511 alloys are approximately 760 MPa (deformed at 9.5 m/s) and 840 MPa (deformed at 9.5 m/s), respectively. Then, the corresponding equivalent stresses are 1320 MPa and 1450 MPa, respectively. By comparison, the equivalent stress for Ti-64 alloy in the present work is 200 MPa lower than that of Rittel’s work (ca. 1500 MPa) [38] and 300 MPa higher than that of Bai’s work (ca. 1020 MPa) [39]. It also should be noted that the value of dynamic failure energy (ca. 80 MJ/m^3^) is about one-fourth of Rittel’s work (ca. 300 MJ/m^3^) [38]. The observed discrepancies can be attributed to forced shear localization in the FHS specimen, and/or difference in the investigated materials.

### 3.3. Microstructural Characterization

The samples for microstructure observation were cut parallel to the deformation direction by electrical discharge machining and metallographic specimens were prepared by standard mechanical polishing and etched in the Kroll’s reagent. Optical microscopy (OM) was performed with LEICA DMI 3000M.

**Figure 13:**
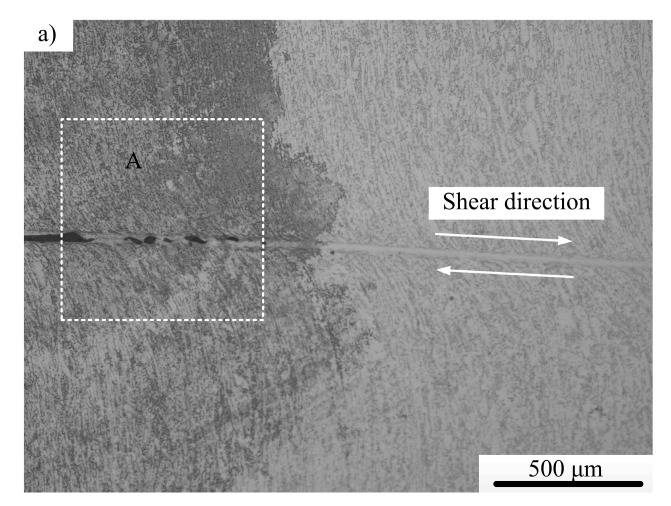

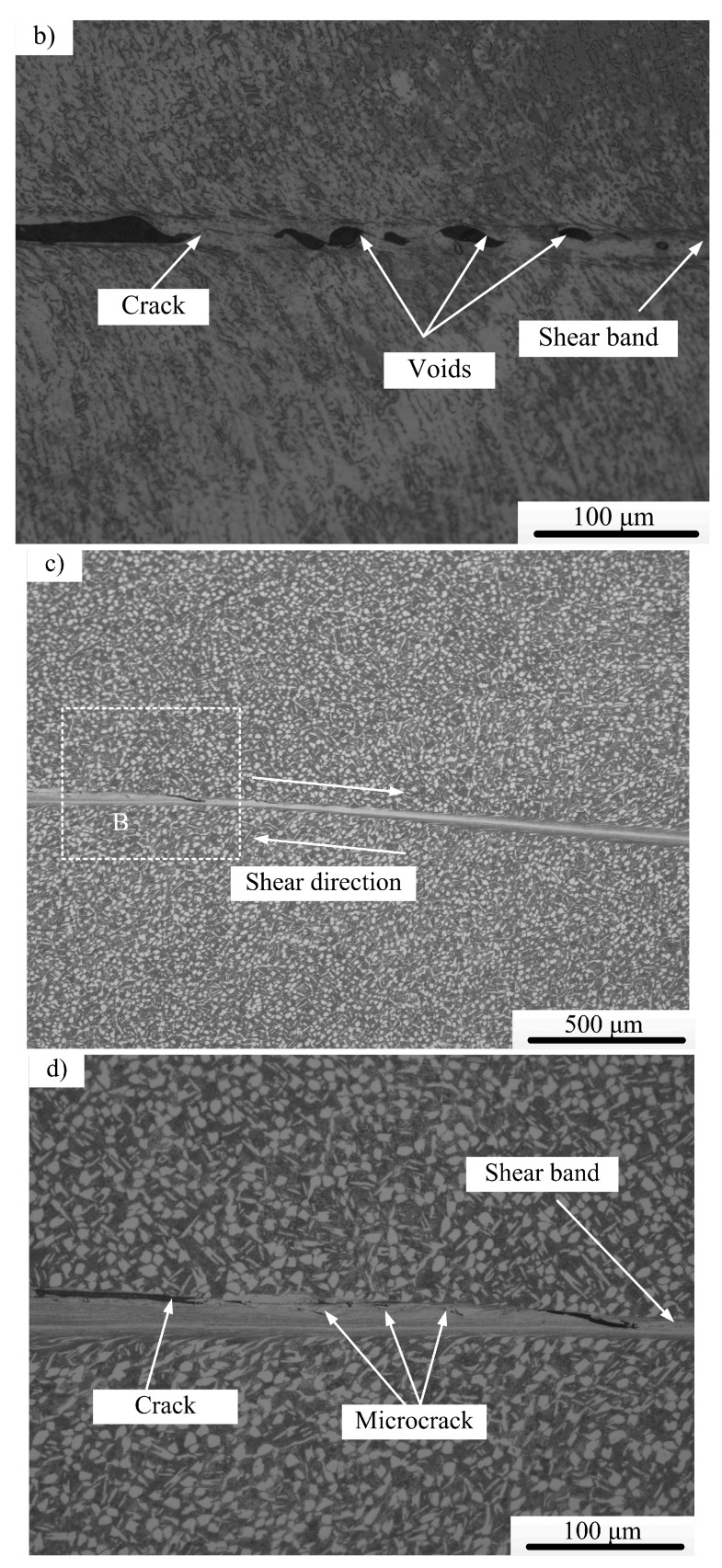
Typical microstructural of (**a**) Ti-64 alloy; (**b**) higher magnification of A in (a); (**c**) Ti-55511 alloy and (**d**) higher magnification of B in (c).

Figure 13 shows the typical microstructural of a well-developed localized shear region (ASB) for Ti-64 and Ti-55511 alloys. The four figures presented in Figure 13 correspond to Ti-64 alloy loaded at 9.2 m/s (Figure 13a), higher magnification of region A in Figure 13a, Ti-55511 alloy loaded at 9 m/s, and higher magnification of region B in Figure 13b, respectively. As shown in Figure 13a,b, elliptical voids form at the left side of the ASB, and a crack forms at the end of the left side. As illustrated in Figure 13c,d, microcracks form at the end of the left side of the ASB.

**Figure 14:**
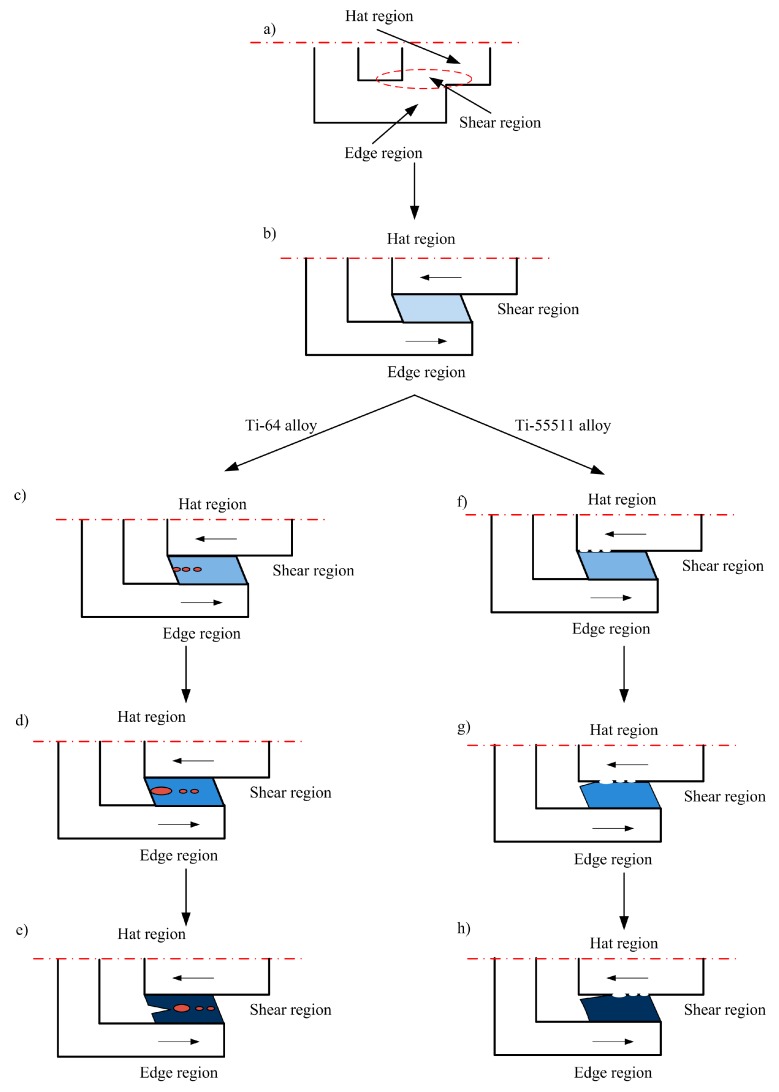
Schematic diagram showing the mechanisms of dynamic failure for two investigated materials. (**a**) undeformed sample; (**b**) ASB formation; (**c**) voids occurred; (**d**) adjoining voids coalesce; (**e**) crack formed and propagated within ASB; (**f**) microcracks formation (white points at the shear band/matrix interface); (**g**) adjoining microcracks coalesce; and (**h**) crack propagated within ASB.

The mechanisms of dynamic failure for the two investigated materials are illustrated in Figure 14. Apparently, two different failure mechanisms can be observed in the shear bands for the two investigated materials. For Ti-64 alloy, ASB forms first (Figure 14b), then elliptical voids initiate at a higher strain localization point within the shear band (Figure 14c). With further deformation, adjoining voids coalesce to bigger voids (Figure 14d) and a crack, and the crack propagates within the ASB up to failure or fracture (Figure 14e). This finding is consistent with the work of Bai et al. [8] and Lee et al. [40]. Apparently, to some extent, the evolution of the ASB in Ti-64 alloy is similar to the features of cohesive fracture. By contrast, for Ti-55511 alloy, ASB forms firstly (Figure 14b), then microcracks form (Figure 14f). Although the microcracks extend into the shear band to some extent, almost all microcracks occurred at the shear band/matrix interface. With further deformation, adjoining microcracks coalesce to a bigger crack (Figure 14g), and the crack propagates within the ASB up to failure or fracture (Figure 14h). This agrees well with the finding from Ran et al. [17] and Wang et al. [41]. Contrary to Ti-64 alloy, the evolution of the ASB in Ti-55511 alloy is similar to the features of adhesive fracture.

Hence, based on the above analysis, the dynamic failure mechanism for Ti-64 alloy can be tentatively identified as cohesive fracture, while the dynamic failure mechanism for Ti-55511 alloy is adhesive fracture. The importance of each failure mechanism (cohesive and adhesive) should be further investigated.

## 4. Conclusions

A series of forced shear tests on FHS specimens for Ti-64 and Ti-55511 alloys was performed by split Hopkinson pressure bar setup. The evolution of shear deformation was monitored by an ultra-high-speed camera (Kirana-05M). According to the experimental findings, the following conclusions can be drawn:

Three distinct stages (homogeneous deformation, heterogeneous deformation and fracture localization and propagation) can be identified using the DIC technique for accurate *γ* measurement.

Based on the analysis of evolution of shear strain field, severe strain localization is the main factor leading to loading/stress drop for the two investigated materials, which means strain localization induces the loss of loading carrying capacity.

The microstructural analysis shows that the dynamic failure mechanisms for Ti-64 and Ti-55511 alloys are cohesive and adhesive fractures, respectively, a distinction that was not emphasized in previous studies.

## Figures and Tables

**Table 1:** Experimental parameters.

Material	No.	Punching Depth *δ*/mm	Maximum Loading/kN	Maximum Shear Stress/MPa	Velocity of the Striker Bar *v*/ms^−1^	ASB (Y/N)
Ti-64	1	0.27	17.50	729	8.8	Y
	2	0.30	17.66	736	9.1	Y
	3	0.32	17.68	737	9.1	Y
	4	0.30	17.73	739	9.2	Y
	5	0.30	17.73	739	9.2	Y
	6	0.31	18.23	760	9.5	Y
Ti-55511	1	0.20	19.32	805	8.8	Y
	2	0.22	19.34	806	8.8	Y
	3	0.21	19.51	813	9.0	Y
	4	0.23	20.03	835	9.2	Y
	5	0.22	20.26	844	9.5	Y

**Table 2:** Loading, displacement, nominal shear strain and shear strain measured by DIC technique for Ti-64 alloy deformed at different time, the velocity of the striker bar is 9.2 m/s.

*t*/μs	*F*/kN	*δ*/mm	*γ_nom._*	*ε_xy_*	*γ_eng._*	*γ_eng._/γ_nom._*	${\dot{\gamma }}_{eng.}$/s^−1^	$\dot{\gamma }$/s^−1^
*t*_0_ = 0	0	0	0	0	0	--	0	3290
*t*_1_ = 10	2.78	0.0164	0.012	0.0060	0.0120	1.024	1200	
*t*_2_ = 14	6.61	0.0389	0.028	0.0123	0.0246	0.885	1760	
*t*_3_ = 17	9.46	0.0597	0.043	0.0162	0.0324	0.760	1910	
*t*_4_ = 20	11.84	0.0789	0.056	0.0205	0.0410	0.728	2050	
*t*_5_ = 26	14.58	0.1069	0.076	0.0287	0.0574	0.752	2210	
*t*_6_ = 31	16.32	0.1263	0.090	0.0368	0.0736	0.816	2370	
*t*_7_ = 36	16.90	0.1440	0.103	0.0489	0.0978	0.951	2720	
*t*_8_ = 40	17.18	0.1573	0.112	0.0548	0.1096	0.975	2740	
*t*_9_ = 46	17.52	0.1774	0.127	0.0613	0.1226	0.968	2670	
*t*_10_ = 53	17.89	0.2002	0.143	0.0691	0.1382	0.966	2610	
*t*_11_ = 58	18.23	0.2162	0.154	0.0768	0.1536	0.995	2650	

**Table 3:** Loading, displacement, nominal shear strain and shear strain measured by DIC technique for Ti-55511 alloy deformed at different time, the velocity of the striker bar is 9 m/s.

*t*/μs	*F*/kN	*δ*/mm	*γ_nom._*	*ε_xy_*	*γ_eng._*	*γ_eng._/γ_nom._*	${\dot{\gamma }}_{eng.}$/s^−1^	$\dot{\gamma }$/s^−1^
*t*_0_ = 0	0	0	0	0	0	--	0	3210
*t*_1_ = 13	7.54	0.0337	0.024	0.0095	0.019	0.789	1460	
*t*_2_ = 20	13.83	0.0806	0.058	0.0185	0.037	0.643	1850	
*t*_3_ = 26	16.87	0.1071	0.077	0.0246	0.0492	0.643	1890	
*t*_4_ = 30	18.45	0.1213	0.087	0.0338	0.0676	0.780	2250	
*t*_5_ = 35	19.16	0.1367	0.098	0.0382	0.0764	0.782	2180	
*t*_6_ = 40	19.70	0.1499	0.107	0.0468	0.0936	0.874	2340	
*t*_7_ = 43	19.84	0.1568	0.112	0.0519	0.1038	0.927	2410	
*t*_8_ = 47	20.03	0.159	0.114	0.0648	0.1296	1.141	2760	
*t*_9_ = 50	19.68	0.1738	0.124	0.0783	0.1566	1.261	3130	
*t*_10_ = 52	19.10	0.1788	0.128	0.0968	0.1936	1.516	3720	
*t*_11_ = 53	18.52	0.1814	0.130	0.1045	0.209	1.613	3940	

**Table 4:** Calculation of dynamic failure energy of FHS specimens.

Materials	Dynamic Failure Energy/MJ·m^−3^					
	No. 1	No. 2	No. 3	No. 4	No. 5	No. 6
Ti-64 alloy	75.1	78.6	87.1	83.4	82.6	83.0
Ti-55511 alloy	51.9	56.9	56.3	61.9	55.9	--
